# Quality Changes of Cold-Pressed Black Cumin (*Nigella sativa* L.), Safflower (*Carthamus tinctorius* L.), and Milk Thistle (*Silybum marianum* L.) Seed Oils during Storage

**DOI:** 10.3390/plants12061351

**Published:** 2023-03-17

**Authors:** Živilė Tarasevičienė, Valdas Laukagalis, Aurelija Paulauskienė, Aldona Baltušnikienė, Edita Meškinytė

**Affiliations:** 1 Institute of Plant Biology and Food Sciences, Agriculture Academy Vytautas Magnus University, Donelaičio Str. 58, 44248 Kaunas, Lithuania; 2 Animal Production Research and Innovation Center, Bioeconomy Research Institute, Agriculture Academy Vytautas Magnus University, Donelaičio Str. 58, 44248 Kaunas, Lithuania; 3 Department of Biochemistry, Lithuanian University of Health Sciences, A. Mickeviciaus Str. 9, 44307 Kaunas, Lithuania

**Keywords:** black cumin, safflower, milk thistle, oil, cold press, fatty acids

## Abstract

Oils derived from non-traditional seeds, such as safflower, milk thistle, and black cumin seeds, have recently grown in popularity. Seed oil is in high demand due to consumer interest in illness prevention and health promotion through healthier diets that include a high concentration of monounsaturated and polyunsaturated fatty acids and antioxidant phenolic components. This study assessed the quality characteristics of cold-pressed seed oil at three unique storage times: at the beginning of the trial (i.e., before storage), after 2 months, and after 4 months. The results of the performed analyses indicate that the acidity of extracted black cumin, safflower, and milk thistle seed oil fluctuates considerably over time. The highest acidity level change was detected for black cumin seed oil, from 10.26% after the extraction to 16.96% after 4 months of storage at 4 °C. Consequently, changes between pre- and post-storage peroxide concentrations were discernible after four months. Peroxide value in milk thistle and safflower seed oils increased by 0.92 meq/kg and 2.00 meq/kg, respectively, during the assessed storage time, while that of black cumin was very high and fluctuated. The storage period substantially affects oxidative changes and the oxidation stability of the oil. Major changes were observed in the polyunsaturated fatty acids in seed oil during storage. The essential changes were detected in the black cumin seed oil odor profile after 4 storage months. Their quality and stability, as well as the nature of the changes that occur during the storage of oil, require extensive investigation.

## 1. Introduction

Oils from non-traditional seeds such as safflower seeds, milk thistle seeds, and black cumin seeds have gained increasing popularity in recent years. Seed oil is in great demand because of consumers’ interest in disease prevention and health promotion via better diets that include a high concentration of monounsaturated and polyunsaturated fatty acids and antioxidant phenolic compounds [1].

Milk thistle (*Silybum marianum* L.) is a southern European native annual or biennial herbaceous plant. For thousands of years, its fruit and seeds have been used as an herbal treatment for liver and biliary problems. Its seed’s contents have antioxidant, anti-atherosclerotic, antihypertensive, anti-obesity, anti-diabetic, anti-inflammatory, and anticancer properties. Thus, milk thistle seed oil is a significant byproduct of silymarin industrial manufacturing. It is abundant in unsaturated fatty acids, particularly linoleic and oleic acids, which are beneficial for human health by reducing arteriosclerosis, diabetes, and cancer [2,3].

Safflower (*Carthamus tinctorius* L.) is an oil crop that dates all the way back to prehistoric times. Its remarkable tolerance for drought and salinity highlights the high level of applicability. Two distinct kinds of oils may be generated, depending on the safflower species’ genetics. Nevertheless, safflower oil has a high proportion of linoleic and oleic acids, which are resistant to oxidation. Safflower oil composition varies as well, depending on the method and methodology used to extract the oil. A cultivar with a high oleic content (>70%) is ideal as a heat-tolerant cooking oil. Its hue ranges from light yellow to golden. In some countries, this oil is in use for margarine, salad dressings, baby formula, and food coating production [4].

The third culture of non-traditional seeds analyzed in this article is from *Nigella sativa* L., which is often known as black cumin and is an annual flowering plant. The seeds of black cumin are particularly interesting since they contain considerable amounts of phytochemicals with antioxidant qualities and health benefits. The seeds have a high concentration of fixed oil, which benefits both human health and nutrition owing to the presence of both major (essential fatty acids) and minor substances (phenolic compounds, tocopherols, and sterols). Tocols (tocopherols and tocotrienols) protect the oil from lipid oxidation and enhance its oxidative stability. Among the minor chemicals, thymoquinone is the main active component of the seeds and has several beneficial characteristics, including antioxidant and anti-inflammatory capabilities [5].

The main factors affecting extraction efficiency and the quality of fixed oils are seed pre-treatment and oil extraction methods. All vegetable oil extraction methods can be divided into conventional (organic solvent extraction, hot and cold pressing) and novel extraction methods (supercritical CO_2_ extraction, ultrasound-assisted and microwave-assisted extractions, pulsed electric field extraction, etc.). New extraction methods allow for a higher oil yield, a decreased solvent consumption, and a reduced extraction time. All possible vegetable oil extraction methods have some merits and demerits; therefore, the oil output, cost effectiveness, and recycling of the extraction solvents as well as the obtained oil quality have to be taken into consideration [6]. Cold oil pressing is a simple, cost effective, and safe (for consumers and the environment) extraction method that can provide very pure, safe, and nutritionally dense oil. Although it is a low-energy method that preserves most bioactive chemicals, its disadvantages include a limited yield [7].

During fixed oil storage, physicochemical changes occur, and the quality of oils deteriorates. Due to growing consumer interest in non-traditional seed oil and the lack of knowledge on their quality and stability as well as the nature of changes that occur during storage, substantial research is required. The storage conditions have a significant impact on the level of oxidative alterations, as well as the activity of antioxidants [7]. Therefore, the aim of this study is to analyze the chemical profile of cold-pressed milk thistle, safflower, and black cumin seed oil, as well as to enrich the accumulated data regarding their quality alterations during the storage period.

## 2. Results and Discussion

### 2.1. Physicochemical Properties of Seeds Oils

The results showed that oil extraction yield after cold press extraction without seed pretreatment was 26.88% for black cumin, 19.20% for milk thistle, and 19.68% for safflower seeds. The oil extraction yield of the seeds was quite low since the undamaged cell wall caused major resistance to the oil extraction not only when using a cold press but also when using a solvent. After the extraction of milk thistle seed oil using the hexane as a solvent and before the extraction of milling seeds, the oil yield was 29.43% [8]. Additionally, a reduced oil yield was observed after cold press extraction from black cumin compared to after solvent extraction; this can was explained by the solvent’s ability to extract oleoresins [9]. Oil content in seeds is very dependent on cool temperatures, salinity, and water stress during the plant’s vegetation; therefore, depending on the seed’s origin, black cumin seed oil yield fluctuates from 13–23% from those of Italian to 40% from those of Iranian origin [9,10].

Initial results before storage indicated that all assessed criteria varied significantly between species, and uncommon aspects could be drowned. The same aspect was noted after carrying out an analysis of the seed oils stored for 4 months (Table 1).

The results of the analysis revealed that the acidity or free fatty acids of the extracted black cumin oil varies significantly from 10.26% after the extraction to 16.96% during all storage periods at 4 °C (Table 1).

The highest change of acidity was recorded in the second month of storage for black cumin seed oil, while the acidity of milk thistle and safflower seed oil statistically significantly increased only after the 4 storage months. A significant increase in acidity of 81.82% was recorded after 4 months of storage in milk thistle seed oil, of 65.40% in black cumin, and of 30.95% in safflower seed oil compared with the acidity before storage. These changes indicate the state of oil degradation. Free fatty acid (as oleic acid %) in Egypt-origin cold-pressed black cumin seeds oil was 11.00, while that in the oil obtained from seeds of Turkish origin was 7.49 [9,11]. According to Gharby et al. [10], the free fatty acid percentage in cold-press-extracted black cumin seeds oil of Moroccan origin was 0.9, while in solvent the percentage extracted was 2.3. These results are also in contrast with other scientists’ outcomes, for example those of Nesrain Farhan et. al. [12], who indicated an average value of 20.65% free fatty acids in such seeds. Enzymatic triacylglyceride hydrolysis and saponification reactions cause the formation of free fatty acids (FFAs) in vegetable oils [10]. Lipase can facilitate the hydrolysis of triacylglycerides and the formation of free fatty acids, which can be easily oxidized to peroxides [13].

During a storage period of 12 weeks at 60 °C, the acidity of black cumin oil increased almost linearly from 3.02 to 7.10 g of oleic acid/100 g, indicating constant triglyceride hydrolysis [14]. The hydrolysis of triglycerides can be accelerated by lipase, and free fatty acids can be oxidized easily and produce peroxides; such kinds of oil cannot be used for human consumption [13].

The same observations can be made by analyzing the data on acid value. The highest increase in acid value was observed in milk thistle and black cumin seed oil, and the least of which was in safflower, respectively, by 79.78, 63.03, and 31.60% compared with the oil acid value before storage. The acid value of milk thistle cold-press-extracted oil before pretreatment was 4.24 mg KOH/g oil [15], while Wiem Meddeb et al. [3] reported that, depending on the variety, the acid value of milk thistle varies from 5.48 to 8.34 mg KOH/g oil. Moreover, scientific data has revealed that during 8 storage weeks at 2 °C, the acid value of safflower cold-press-extracted oil increased from 1.39 to 2.57 mg KOH/g oil [7].

High peroxide values show that oils are unstable and have low quality, and they influence the oil’s off flavor [13].

The peroxide values of the different seed oils during storage varies compellingly as well. The highest peroxide value was observed in black cumin seed oil, while the least was in milk thistle (Table 1). The peroxide value of cold-press-extracted milk thistle seed oil depending on the variety fluctuates from 2.83 to 4.20 meq/kg [3]. Therefore, differences in the comparisons between peroxide values before storage and after 4 months of storage indicate changes as follows: peroxide values in milk thistle and safflower increased by 0.92 meq/kg and 2.00 meq/kg, respectively. However, there was a significant decrease in peroxides in black cumin seed oil by 83.67 meq/kg at the same time. Meanwhile, peroxide value showed an equilibrium between some opposite mechanisms, such as oxidation, leading to the peroxide’s formation as well as the degradation of peroxide [16]. The reduction in peroxide value after 4 storage months shows the degradation of peroxide. A slight decrease from 86.32 to 84.72 meq/kg of peroxide value after 4 storage months in black cumin seeds oil was observed by Oubannin et. al. [14]. According to Joana Banas [7] the peroxide value of safflower seed oil over 8 weeks of storage increased from 4.57 to 13.10 meq/kg.

The induction period is a good indicator of the oil susceptibility to oxidation and its shelf life [10]. Curves of the seed oil’s induction period throughout the course of storage duration revealed species-specific differences in the chemical processes. The longest induction period before storage was detected of black cumin seeds oil, while the shortest of which was of safflower oil. The milk thistle seed oil induction period after two storage months significantly decreased, while after 4 storage months it returned to the baseline before storage. Safflower seed oil showed a significant decrease in the oil induction period during all storage periods (Table 2).

During storage, the highest induction period decrease was observed in black cumin seed oil (74.11%) after 2 storage months and significant increased by 138.69% after 4 months compared to after an induction period of 2 months (Table 2). Since the cold-pressed oil was not filtered, it is likely that the release of some sediment compounds in the oil led to an increase in the induction time after two months of storage in milk thistle and safflower, and four months in black cumin oil; therefore, the induction time increased.

According to Chandrasekar et al., [17] antioxidants in oils can inhibit lipid oxidation by quenching free radicals, while pro-oxidants (enzymes, transitional metal ions, photosensitizers) accelerate oxidation through various mechanisms during storage as well as during processing [17].

Depending on the variety, the oil stability index of milk thistle fluctuated from 4.55 to 8.75 h [3]. An induction time of 13 and 9 h was found for cold press- and solvent-extracted *Nigella sativa* seed oils, respectively [10]. Soxhlet-extracted and microwave-assisted extracted black cumin seed oil had the highest induction time, respectively (19.6 and 18.4 h), while the cold-pressed black seed oil had just 3.48 h [13]. A sufficient number of scientific research has shown that the phenolic compounds of plant oil have a considerable effect on the oxidative stability of these products. Nevertheless, it is not always possible to associate a higher amount of phenolic compounds with better oxidative properties, since different phenolic compounds have different effects on the oxidative stability of the oil. Therefore, further studies would provide a better understanding of the mechanism of their protective action on oils, particularly under natural storage [18].

The content of minor compounds such as polyphenols, tocopherols or phospholipids can explain induction time variability [8]. In the evaluated seed oils, relatively modest levels of phenolic compounds have been found. Even though considerable variances have been discovered in both sections—storage duration and species—they are not identical. Even while the highest concentration of phenolic compounds was detected in black cumin seed oil at 140.19 mg 100 g^−1^ at the beginning of storage and 107.96 mg 100 g^−1^ after 4 months of storage, this decrease was statistically significant. The same tendency was detected in safflower seeds oil, wherein total phenol content decreased during oil storage by 94.29, and in milk thistle it decreased by 20.65 mg 100 g^−1^. Pearson correlation coefficients between the amount of total phenols and oil induction time were calculated, and a statistically significant relationship was observed only in *Nigella sativa* seeds oil (r = 0.9988).

### 2.2. Seeds Press Cake Chemical Content

The chemical content of seed press cake is provided in Table 2. The highest dry matter content was detected in milk thistle seed press cake, while the lowest of which was found in safflower—91.67% and 88.81%, respectively.

**Table 2:** Seeds press cake chemical content.

Parameter	Milk Thistle	Safflower	Black Cumin
Dry matter, %	91.67 ± 0.55 ^a^	88.81 ± 0.06 ^c^	89.56 ± 0.10 ^b^
Protein, %	24.54 ± 0.03 ^a^	23.20 ± 1.00 ^b^	24.76 ± 0.11 ^a^
Fiber, %	38.30 ± 2.50 ^b^	43.09 ± 0.50 ^a^	12.66 ± 0.10 ^c^
Fats, %	8.24 ± 0.14 ^c^	9.78 ± 0.94 ^b^	23.24 ± 0.53 ^a^
Ash, %	5.58 ± 0.06 ^a^	3.91 ± 0.17 ^b^	5.67 ± 0.11 ^a^
Phenolic compounds, mg GAE 100 g^−1^	0.07 ± 0.01 ^b^	0.08 ± 0.01 ^b^	1.44 ± 0.01 ^a^

Lowercase letters “^a,b,c^” indicates significant differences when *p* < 0.05 (i.e., 95% reliability) between species.

When comparing the findings of the protein content of seed oil cakes, black cumin and milk thistle had the highest concentrations—24.76 and 24.54%, respectively. At 23.20%, a significant difference was seen in comparison to safflower. The amount of fiber was the highest in safflower seed press cake. The least amount of that chemical component was detected in black cumin seed press cake—just 12.66%. Similar outcomes were noted in Mohamed F. R. Hassanien et al. [19]’s research, which had a result of 8.40%.

When examining various fat concentrations in the samples of seed oil cakes, a noticeable, statistically significant difference was seen across all assessed species. The lowest quantity of fats was found in milk thistle at 8.24%, while the largest amount was found in black cumin at 23.24%.

The highest amount of ash was found in black cumin and milk thistle,—5.67 and 5.58%, respectively, while the lowest was found in safflower at 3.91%. In Mohamed F. R. Hassanien et al. [20]’s research, ash amount in black cumin seed oil was 4.80%.

In terms of phenolic components, milk thistle and safflower seed oil cakes did not differ significantly from one another. In addition, the average amount of identified phenolic chemicals was just 0.08 mg 100 g^−1^. Meanwhile, the largest quantity was identified in black cumin seed oil cake at 1.44 mg 100 g^−1^.

Evidently, safflower seed oil cake has a substantial quantity of fiber, which should be evaluated further for its adaptability to a healthy diet. In the meantime, black cumin seed oil cake can be regarded as the seed capable of retaining large concentrations of phenolic components in both seed oil and seed oil cake. Thus, more studies should be conducted to investigate its pharmacological use.

### 2.3. Percentage of Fatty Acid Groups in Seeds Oils from a Total Fatty Acid Content

Fatty acids for edible oil are one of the crucial parameters determining the quality of oils. The fatty acid composition of different oily seeds is presented in Table 3 and Table 4. Storage time essentially influenced the composition of the oils’ fatty acids. The highest amount of saturated fatty acids before storage was detected in milk thistle and safflower seed oil; meanwhile, it was the least in black cumin seeds oil and differed significantly compared to the others investigated seed oils.

According to Bahram Fathi-Achachlouei et al. [8], milk thistle seed oil possessed a low saturated fatty acid content (19.41%); likewise, Z.S. Zhang, et al. found this measurement to be 22.06% [2]. S. Oubannin et al. [14] revealed an almost two times lower content of SFA in black cumin originating from Morocco compared to that which was observed in our study; theirs consisted of 15.45%. Dabbour et al. [1] determined that the SFA content of cold-pressed milk thistle seed oil was 19.53%, while that of MUFA and PUFA was 22.92% and 57.55%, respectively.

Storage resulted in a decrease in saturated fatty acids in milk thistle seed oil (Table 3). A decrease in SFAs by 3.05 percent was observed after storage compared to their amount before the storage in milk thistle seed oil (Table 3).

Prior to storage, the total amount of MUFAs differed significantly in all investigated seed oils. A storage period of four months resulted in an increase in MUFAs in milk thistle and black cumin seed oil, while in safflower, this did not change significantly.

The highest amount of polyunsaturated fatty acids was observed in black cumin seed oil. There was detected a significant reduction in polyunsaturated fatty acids during storage for safflower and black cumin seed oil, while in milk thistle, the percentage of PUFAs was stable.

Safflower and black cumin went from 36.93% and 41.79% before storage to 34.54% and 39.44%, respectively, after 4 months of storage. In addition, the highest decrease was observed in safflower seed oil—2.39%.

Before storage, the amount of omega-3 acid differed in all species’ seed oil, while the highest amount was in black cumin, and the least was in milk thistle seed oil. A significant decrease was observed in milk thistle and safflower seed oil after four storage months. A storage period of four months resulted in the stability of omega-6 acids in all the species evaluated, with the highest amount being in black cumin seed oil before the storage and that of milk thistle after storage.

The amount of omega-9 fatty acids before storage was significantly different in all investigated seed oil. In black cumin seed oil, it was almost two times higher than in safflower. Significant increases in omega-9 acids have been seen, particularly in milk thistle, where the amount of these fatty acids after 4 months of storage increased by 30.82%. However, safflower oil exhibited an increase in omega-9 acid by 12.70%, while that in black cumin seed oil didn’t change significantly.

The MUFA/PUFA ratio fluctuated depending on the seed’s species. Overall, it was susceptible to increase during the storage of oil in black cumin by 0.10, in safflower oil by 0.07, and in milk thistle by 0.02.

The PUFA/SFA ratio can be used to observe the tendency of oil autoxidation [21]. The PUFA/SFA ratio is one of the main parameters currently used to assess the nutritional quality of the lipid fraction of foods and is recommended to be >0.4 [22]. This ratio in our analyzed oils before storage was 1.69, 1.15 and 1.13 in black cumin, safflower, and milk thistle, respectively (Table 3). The storage period changed the PUFA/SFA ratio in the investigated seed oils, and it was observed to increase the ratio in all investigated seed oils by 0.16, 0.13 and 0.13 in milk thistle, safflower and black cumin, respectively.

The highest change in the n6/n3 ratio during storage was observed in milk thistle and safflower seed oil, where the ratio increase almost doubled, while in black cumin there was a slight decrease.

The unsaturated/saturated fatty acid ratio during storage was unstable. It was determined that the ratio decreased in safflower and black cumin oil, while in milk thistle oil it increased (Table 3).

### 2.4. Percentage of Fatty Acids in Seeds Oils from a Total Fatty Acid Content

The milk thistle, safflower, and black cumin seed oils were evaluated for a total of 36 fatty acids before and after storage (further details provided in Table 4).

The predominant fatty acid in milk thistle seed oil before the storage was palmitic, linoleic, linolelaidic, *tr*-9-elaidic, and *cis*-9-oleic acid at 15.60, 14.48, 11.54, 10.56, and 8.24%, respectively (Table 4). Hence, the predominant fatty acid was linoleic and oleic, with two isomers: *tr*-9-elaidic, and *cis*-9-oleic acid. Bahram Fathi-Achachlouei et al. [15,23]’s research showed that linoleic acid (18:2n-6) was the prominent fatty acid, followed by oleic acid (18:1n-9), palmitic acid (C16:0), and stearic acid (18:0). The fatty acid composition of milk thistle oil depends on the geographical location and seed origin. In milk thistle seeds oil native to Egypt, researchers reported 53.3% linoleic, 20.8% oleic, 9.4% palmitic, and 6.6% stearic acids [19], while in Iranian milk thistle, measurements were 49.7–53.6% linoleic, 22.8–28.9% oleic, 7.3–8.4% palmitic, and 4.6–6.8% stearic acid [15], and in Tunisia, the reported content of linoleic, oleic, palmitic and stearic acid was 59.98, 21.26, 12.74, and 3.24%, respectively [24]. In our investigation the amount of stearic acid in milk thistle before the storage was 4.59%.

As for safflower, it had the highest amount of linoleic, palmitic, *tr*-9-elaidic, *cis*-9-oleic, and stearic acids at 23.00, 14.89, 12.55, 9.27, 8.18%, respectively, before the storage. According to Aydeniz et al. [25] safflower oil is the oil that contains the highest amount of linoleic acid among all commercial oils. Researchers’ data indicate that safflower oil consists of 71–75% linoleic, 16–20% oleic, 6–8% palmitic, and 2–3% stearic acid. Topkafa [26] indicates that PUFAs are the main fatty acids (73.8%) in safflower oil, with the highest amount of linoleic acid (73.7%) from the total fatty acids. The lower amount of linoleic acid in safflower seed oil was determined by Celenk, Gumus¸, Argon, Buyukhelvacigil, and Karasulu [27]; they reported 58.2% linoleic acid in the oil. Recently, a safflower breeding effort has led to high oleic fatty acids in safflower hybrid selections [28]. These plants are more suitable for biobased applications (such as the production of biolubricants, bioherbicides, bioplastics, etc.) rather than for food needs [29]. The quality of oil is defined by the content of linoleic and oleic acids. High oleic oils have increased oxidation stability and have indicate higher quality [30]. The variation in safflower seed oil’s fatty acids depending on growing region was introduced by the research of Federica Zanetti [31]. SFA and MUFA were 6% and 5% higher, respectively (*p* ≤ 0.05) in the Emilia-Romagna region than in Tuscany, while PUFA content showed great variability across the growing regions, with a remarkable increase of 38% when safflower was grown in Tuscany compared to in Emilia-Romagna [31].

In black cumin seed oil, the predominant fatty acids before storage were linoleic, palmitic, *tr*-9-elaidic, *cis*-9-oleic, and linolelaidic acids at 19.66, 15.12, 12.62, 11.87, and 11.75%, respectively. S. Oubannin [14] reported that the predominant fatty acid in cold-pressed black cumin seed oil is linoleic, oleic, and palmitic acid at 58.06, 23.14, and 12.05%, respectively, while in oil obtained by Soxlet extraction, the amount of linoleic acid was 57.71%, oleic was 24.46%, palmitic was 12.17%, eicosadienoic was 2.52%, and stearic acid was 2.31% [32]. Depending on the seed’s origin, the oil extraction method’s C18:2 fatty acid fluctuated from 47.5 to 62.4%, C18:1 from 12.7 to 25.0% and C16:0 from 8.59 to 15.0% [13].

During storage, significant changes in the content of fatty acids occur, which depend on oil extraction methods and storage conditions. Oil oxidation causes the main oil changes, and it is a complex process with two different mechanisms, i.e., autoxidation and photosensitized oxidation (photo-oxidation). The initialization of autoxidation requires lipid radicals, while for photo-oxidation it requires a photosensitizer, such as chlorophyl [33]. Even though several environmental factors act during oil storage, such as light and oxygen, an increase in temperature leads in an increase to autooxidation and the decomposition of hydroperoxides. As a result, the undesirable flavors of oxidized oil occur [34]. The main change in storing oil occurs due to the decrease in PUFAs.

Palmitic acid content in milk thistle oil decreased by 18.8%, in black cumin it increased by 6.7%, and it did not change significantly in safflower oil (Table 4). The same tendency in milk thistle and safflower oil was observed concerning the changes in stearic acid during storage. Stearic acid in the milk thistle seed oil increased by 45.8%, and in safflower oil, it increased by 16.5%, while in black cumin it was stable during storage. Behenic acid in the milk thistle oil showed a decrease of 26.9%. In terms of *tr*-9-elaidic acid, an increase was observed in milk thistle, safflower, and black cumin seeds oils by 38.3%, 12.2%, and 18.3%, respectively. Other monounsaturated fatty acids and the *cis*-9-oleic amount in milk thistle and safflower increased by 30.2% and 15.3%, respectively, while in black cumin it did not change significantly.

More significant changes in fatty acids content were determined in terms of polyunsaturated fatty acids. The main changes were observed in the content of linoleic, linolelaidic, linolenic, arachidonic acids. In milk thistle, the amount of linoleic acid increased by 10.8% and in safflower by 6.6%, but in black cumin, no significant changes were observed. Analyzing linolelaidic acid content, for instance, revealed a consistent rise in milk thistle by 30.6% and in safflower by 6.5%. Otherwise, the amount of linolelaidic acid in black cumin did not change significantly. The reduction was particularly noticeable in the content of linolenic acid. An almost two-fold decrease in linolenic fatty acid content in milk thistle and safflower was observed, but significant changes were not observed terms of black cumin. We observed a decrease in arachidonic acid in milk thistle by 35.5% and in safflower by 12.6%, but in black cumin this measure was stable (Table 4).

**Table 4:** Percentage of fatty acids in seeds oils from a total fatty acid content.

Systematic (Trivial) Name	Milk Thistle	Safflower	Black Cumin
*Saturated Fatty Acids*			
Butanoic (Butyric) acid, %			
Before storage	0.01 ± 0.00 ^b^	Not detected	0.63 ± 0.01 ^b^
4 months after storage	0.02± 0.00 ^a^	Not detected	0.76 ± 0.00 ^a^
Hexanoic (Caproic) acid, %			
Before storage	0.01 ± 0.00 ^a^	0.02 ± 0.00 ^a^	0.03 ± 0.00 ^a^
4 months after storage	0.01 ± 0.00 ^a^	0.02 ± 0.00 ^a^	0.03 ± 0.00 ^a^
Octanoic (Caprylic) acid, %			
Before storage	0.02 ± 0.00 ^a^	0.02 ± 0.00 ^a^	0.02 ± 0.00 ^a^
4 months after storage	0.02 ± 0.00 ^a^	0.02 ± 0.00 ^a^	0.02 ± 0.00 ^a^
Decanoic (Capric) acid, %			
Before storage	0.03 ± 0.00 ^a^	0.02 ± 0.00 ^a^	0.02 ± 0.00 ^b^
4 months after storage	0.02 ± 0.00 ^a^	0.02 ± 0.00 ^a^	0.02 ± 0.00 ^a^
Undecanoic acid, %			
Before storage	0.01 ± 0.00 ^b^	0.02 ± 0.00 ^a^	0.02 ± 0.00 ^a^
4 months after storage	0.01 ± 0.00 ^a^	0.02 ± 0.00 ^a^	0.01 ± 0.00 ^a^
Dodecanoic (Lauric) acid, %			
Before storage	0.05 ± 0.00 ^a^	0.04 ± 0.00 ^a^	0.05 ± 0.00 ^a^
4 months after storage	0.03 ± 0.00 ^b^	0.03 ± 0.00 ^a^	0.04 ± 0.00 ^a^
Tridecanoic (Tridecylic) acid, %			
Before storage	0.01 ± 0.00 ^a^	0.01 ± 0.00 ^a^	0.01 ± 0.00 ^a^
4 months after storage	0.01 ± 0.00 ^a^	0.02 ± 0.00 ^a^	0.01 ± 0.00 ^a^
Tetradecanoic (Myristic) acid, %			
Before storage	0.06 ± 0.00 ^a^	0.04± 0.00 ^a^	0.10 ± 0.01 ^a^
4 months after storage	0.03 ± 0.00 ^b^	0.05 ± 0.00 ^b^	0.07 ± 0.00 ^a^
Pentadecanoic (Pentadecylic) acid, %			
Before storage	0.26 ± 0.01 ^a^	0.44 ± 0.01 ^a^	0.24 ± 0.01 ^a^
4 months after storage	0.13 ± 0.00 ^b^	0.41 ± 0.00 ^a^	0.20 ± 0.01 ^a^
Hexadecanoic (Palmitic) acid, %			
Before storage	15.60 ± 0.15 ^a^	14.89 ± 0.65 ^a^	15.12 ± 0.29 ^b^
4 months after storage	12.67 ± 0.06 ^b^	15.73 ± 0.06 ^a^	16.13 ± 0.12 ^a^
Heptadecanoic (Margaric) acid, %			
Before storage	0.61 ± 0.01 ^a^	0.74± 0.02 ^a^	0.41 ± 0.01 ^a^
4 months after storage	0.36 ± 0.00 ^b^	0.61 ± 0.00 ^b^	0.38 ± 0.01 ^a^
Octadecanoic (Stearic) acid, %			
Before storage	4.59 ± 0.02 ^b^	8.18± 0.19 ^b^	5.85 ± 0.09 ^a^
4 months after storage	8.47 ± 0.08 ^a^	9.53± 0.03 ^a^	5.83 ± 0.06 ^a^
Eicosanoic (Arachidic) acid, %			
Before storage	Not detected ^a^	Not detected	0.49 ± 0.03 ^a^
4 months after storage	0.02 ± 0.02 ^a^	Not detected	0.58 ± 0.01 ^a^
Heneicosanoic acid, %			
Before storage	0.45 ± 0.01 ^a^	0.31± 0.00 ^a^	0.06 ± 0.00 ^a^
4 months after storage	0.26 ± 0.01 ^b^	0.26± 0.00 ^b^	0.05 ± 0.00 ^b^
Docosanoic (Behenic) acid, %			
Before storage	7.36 ± 0.20 ^a^	4.08 ± 0.05 ^a^	0.44 ± 0.02 ^a^
4 months after storage	5.38 ± 0.04 ^b^	3.98 ± 0.01 ^a^	0.34 ± 0.01 ^a^
Tricosanoic (Tricosylic) acid, %			
Before storage	0.55 ± 0.02 ^a^	0.59 ± 0.01 ^a^	0.12 ± 0.01 ^a^
4 months after storage	0.34 ± 0.00 ^b^	0.53 ± 0.01 ^b^	0.08 ± 0.00 ^a^
Tetracosanoic (Lignoceric) acid, %			
Before storage	4.09 ± 0.13 ^a^	2.83 ± 0.03 ^a^	0.37 ± 0.02 ^a^
4 months after storage	2.87 ± 0.03 ^b^	2.80 ± 0.02 ^b^	0.28 ± 0.01 ^a^
*Monounsaturated fatty acids*			
Eicosane acid, %			
Before storage	5.69 ± 0.24 ^a^	2.52 ± 0.01 ^a^	1.15 ± 0.06 ^a^
4 months after storage	2.35 ± 0.02 ^b^	1.11 ± 0.00 ^b^	1.29 ± 0.08 ^a^
*cis*-9-Tetradecenoic (Myristoleic) acid, %			
Before storage	1.00 ± 0.01 ^a^	1.53 ± 0.01 ^b^	1.09 ± 0.05 ^a^
4 months after storage	0.55 ± 0.00 ^b^	1.58 ±0.00 ^a^	0.91 ± 0.01 ^a^
*cis*-9-Hexadecenoic (Palmitoleic) acid, %			
Before storage	0.87 ± 0.01 ^a^	1.39 ± 0.04 ^a^	5.64 ± 0.02 ^b^
4 months after storage	0.51 ± 0.00 ^b^	1.58 ± 0.00 ^a^	6.95 ± 0.05 ^a^
*cis*-10-pentadecenoic acid, %			
Before storage	0.21 ± 0.00 ^a^	0.08 ± 0.00 ^a^	0.12 ± 0.00 ^b^
4 months after storage	0.11 ± 0.00 ^b^	0.04± 0.01 ^a^	0.14 ± 0.00 ^a^
*cis*-10-heptadecanoic acid, %			
Before storage	0.42 ± 0.01 ^a^	0.61 ± 0.00 ^a^	0.41 ± 0.01 ^a^
4 months after storage	0.22 ± 0.00 ^b^	0.54 ± 0.01 ^b^	0.33 ± 0.00 ^a^
*cis*-13-Docosenoic (Erucic) acid, %			
Before storage	0.48 ± 0.02 ^a^	0.34 ± 0.01 ^a^	0.61 ± 0.07 ^a^
4 months after storage	0.28 ± 0.00 ^b^	0.28 ± 0.00 ^b^	0.19 ± 0.00 ^b^
*cis*-15-Tetracosenoic (Nervonic) acid, %			
Before storage	0.61 ± 0.02 ^a^	2.57± 0.03 ^b^	Not detected
4 months after storage	0.41 ± 0.00 ^b^	2.77 ± 0.01 ^a^	Not detected
*cis*-9-oleic acid, %			
Before storage	8.24 ± 0.45 ^b^	9.27 ± 0.12 ^b^	11.87 ± 0.18 ^a^
4 months after storage	10.73 ± 0.21 ^a^	10.69 ± 0.18 ^a^	10.83 ± 0.28 ^a^
*tr*-9-elaidic acid, %			
Before storage	10.56 ± 0.12 ^b^	12.55 ± 0.01 ^b^	12.62 ± 0.01 ^b^
4 months after storage	14.60 ± 0.15 ^a^	12.86 ± 0.03 ^a^	14.93 ± 0.12 ^a^
*Polyunsaturated fatty acids*			
6,9,12-Octadecatrienoic (g-linolenic) acid, %			
Before storage	0.71 ± 0.03 ^a^	0.86± 0.02 ^a^	0.98 ± 0.02 ^a^
4 months after storage	0.02 ± 0.00 ^b^	0.82 ± 0.00 ^a^	0.03 ± 0.01 ^b^
9,12-Octadecadienoic (Linoleic) acid, %			
Before storage	14.48 ± 0.08 ^b^	23.00 ± 0.13 ^b^	19.66 ± 0.79 ^a^
4 months after storage	16.04 ± 0.10 ^a^	24.51 ± 0.06 ^a^	16.69 ± 0.13 ^a^
*cis*-5,8,11,14,17-Eicosapentaenoic (Eicosapentaenoic) acid, %			
Before storage	0.26 ± 0.01 ^a^	0.58± 0.00 ^b^	0.53 ± 0.03 ^a^
4 months after storage	0.02 ± 0.02 ^b^	0.50 ± 0.02 ^a^	0.53 ± 0.00 ^a^
*cis*-8,11,14-eicosatrienoic (Dihomo-g-linolenic) acid, %			
Before storage	0.59 ± 0.03 ^a^	3.79± 0.03 ^b^	1.36 ± 0.01 ^a^
4 months after storage	0.27 ± 0.01 ^b^	1.89 ± 0.02 ^a^	1.09 ± 0.01 ^b^
*cis*-11,14-eicosadienoic (Eicosadienoic) acid, %			
Before storage	1.14 ± 0.70 ^b^	1.26 ± 0.02 ^a^	4.55 ± 0.14 ^a^
4 months after storage	1.29 ± 0.01 ^a^	0.61 ± 0.01 ^b^	4.44 ± 0.04 ^a^
Linolelaidic acid, %			
Before storage	11.54 ± 0.08 ^b^	3.27 ± 0.29 ^a^	11.75 ± 0.29 ^a^
4 months after storage	15.07 ± 0.02 ^a^	3.07 ± 0.01 ^b^	13.51 ± 0.37 ^b^
9,12,15-Octadecatrienoic (Linolenic) acid, %			
Before storage	0.74 ± 0.02 ^a^	0.46 ± 0.01 ^a^	2.10 ± 0.00 ^b^
4 months after storage	0.49 ± 0.00 ^b^	0.20 ± 0.00 ^b^	2.18 ± 0.02 ^a^
*cis*-5,8,11,14-eicosatetraenoic (Arachidonic) acid, %			
Before storage	8.51 ± 0.19 ^a^	3.22 ± 0.00 ^a^	1.21 ± 0.05 ^a^
4 months after storage	6.28 ± 0.05 ^b^	2.86 ± 0.03 ^b^	1.06 ± 0.01 ^a^
*cis*-4,7,10,13,16,19-docosahexaenoic (Docosaheptaenoic) acid, %			
Before storage	Not detected	0.35 ± 0.01 ^a^	Not detected
4 months after storage	Not detected	0.04 ±0.01 ^b^	Not detected
*cis*-13,16-docosadienoic acid, %			
Before storage	0.26 ± 0.00 ^a^	0.15 ± 0.01 ^a^	0.37 ± 0.04 ^a^
4 months after storage	0.15 ± 0.00 ^b^	0.06 ± 0.00 ^b^	0.38 ± 0.01 ^a^

Lowercase letters “^a,b,c^” indicate significant differences in storage time when *p* < 0.05 (i.e., 95% reliability).

Linoleic and linolenic acids and long-chain PUFAs are the main unsaturated fatty acids involved in the oxidation process, which lead to the formation of the compounds that give a product an unpleasant odor, and it also causes toxic biological activity [35].

**Figure 1:**
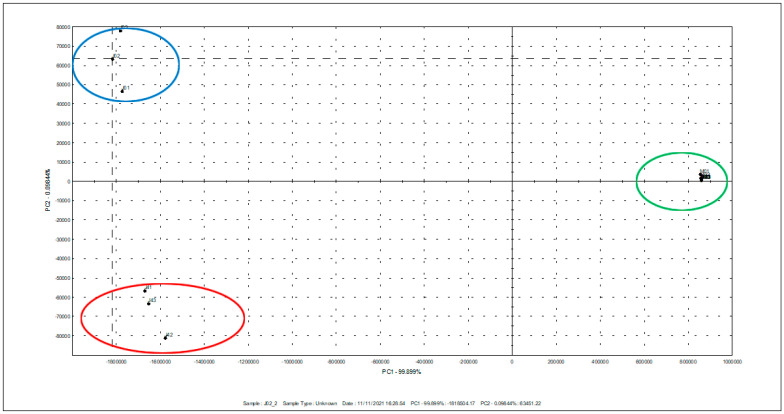
Principal component analysis (PCA) for organic volatile compounds in milk thistle, safflower, and black cumin seed oils during storage of 4 months.

As can be seen in Figure 1, a separation of the oil samples clearly occurs in the case of black cumin seed oil during storage. However, the samples of milk thistle and safflower oils were situated at the positive PC1 values (marked in green color), while the samples of black cumin before storage were at the positive PC2 (marked in blue color), and after 4 storage months, they were at the negative PC1 (marked in red color). The aroma profile of the black cumin seed oil differed extensively compared to the other investigated seed oils. The tentative fixed oil compounds were presented in Table S1.

## 3. Materials and Methods

### 3.1. Materials

Seeds of black cumin (*Nigella sativa* L.) originating from Egypt and safflower (*Carthamus tinctorius* L.) and milk thistle (*Silybum marianum* L.) originating from Poland were obtained from a local oil processor. All the chemicals used in the analysis were of analytical grade and obtained from standard commercial suppliers.

### 3.2. Oil Extraction

Press extraction was carried out using cold press Comet D85 1G (IBG Monforts Oekotec GmbH & Co, Mönchengladbach, Germany) at room temperature without any seed pre-treatment. Oil was stored in dark bottles at 4 °C temperature until the analyses.

### 3.3. Physicochemical Oil Parameters

Free fatty acid and acidity were determined according to LST EN ISO 660:1996 [36], and peroxide value according to LST EN ISO 3960:2001 [37].

### 3.4. Oxidative Stability of Oil

The oxidative stability of oil was determined according to LST ISO 6886:2016 [38] and the induction period (h) was recorded by a 743 Rancimat (Metrohm, Herisau, Switzerland) apparatus by accelerated oxidation test.

### 3.5. Total Phenols Content of Oil

Briefly, the oil sample was diluted with hexane and triple-extraction was performed with water:methanol (80:20, *v*/*v*) following the centrifugation. The total phenol content of the extracts was determined according to the Folin–Ciocalteu spectrophotometric method at 765 nm using a gallic acid calibration curve [39].

### 3.6. Fatty Acids of Oil

The fatty acid composition analysis was based on LST EN ISO 12966-2:2011 [40] and LST EN ISO 15304:2003 [41]. To prepare the fatty acid methyl esters (FAME), fatty acids were extracted using a 20 mL n-heksane and saponified with a 2M KOH methanol solution. The fatty acid composition was analyzed using a Shimadzu GC-2010 gas chromatograph. It was equipped with a BPX-70, 120 m capillary column. Nitrogen was used as a carrier gas. The split ratio was 1:30. The injection temperature was 250 °C. The oven temperature was programmed according to the following sequence: 2 min at 60 °C and increasing up to 230 °C at 20 °C/min and 45 min at 230 °C. Detector temperature was 270 °C. The ionization voltage was 70 eV; the scanning range was 50–550 m/z. “Supelco 37 Component FAME Mix” (Sigma-Aldrich) was used as a standard for FFA identification, and C13:1 tetradecadiene (C14:2) and hexadecadiene (C16:2) fatty acids were identified by interpolation.

### 3.7. Volatile Compounds of Oil

The Heracles II electronic nose (Alpha M.O.S., Toulouse, France) based on ultrafast gas chromatography was applied to analyze the volatile compounds of the fixed oils according to the method described by Wojtasik-Kalinowska et. al. [42]. Briefly, 1 g of oil was placed in glass vials (20 mL) and capped with a Teflon-faced silicon rubber cap. The vials were placed in the automatic sampler. Each vial was incubated at 50 °C for 10 min under agitation at 500 rpm. The accumulated gas in the headspace was then injected into GC with 10 m lengths, 0.18 mm internal diameter two different polarity columns, non-polar MXT-5 (5% diphenyl) and semi polar MXT-1701 (14% cyanopropylphenyl), with two flame ionization detectors (FID). The injected volume was 2500 µL, and the injector temperature was 200 °C. The temperature of the two flame ionization detectors was 270 °C. The injection on the e-nose was carried out on 3 replicates. The method was calibrated using an alkane solution (n-butane to n-hexadecane) in order to convert retention time in Kovats indices and to identify the volatile compounds using the AroChemBase database.

### 3.8. Seeds Press Cake Chemical Content

The dry matter content of the press cake was determined by drying the sample to the constant weight at 105 °C, the amount of ash by burning the sample in muffle at 550 °C, fiber content according to LST EN ISO 6865:2001 [43], protein content by Kjeldahl (LST EN ISO 5983-1:2005/AC:2009) [44], and the amount of fat by Soxhlet extraction with petrol ether. The total phenol content was determined according to the Folin–Ciocalteu spectrophotometric method at 750 nm using a gallic acid calibration curve [45]. The results were expressed as mg of gallic acid in 100 g of sample.

### 3.9. Statistical Methods

The data obtained from three replications were analyzed by one-way analysis of variance (ANOVA) using Statistica software (Statistica 12; StatSoft, Inc., Tulsa, OK, USA). Differences among the means were compared using Fisher’s post-hoc test at a significance level of 0.05. The Person correlation coefficient was calculated in order to determine the relationship between the amount of total phenols and the induction period of oil at a significance level of 0.05. Aiming to assess the impact of storage duration on the aroma profile of fixed oils, a principal component analysis (PCA) of the oils’ volatile compounds was conducted using the Alpha M.O.S. Heracles II device.

## 4. Conclusions

Overall, the data collected from this research resulted in the following conclusions.

The black cumin, milk thistle, and safflower seeds had relative high oil extraction yields following cold press extraction without pretreatment of the seeds. The highest value indicated in black cumin extraction was a yield of 26.88%.

The acidity of the extracted black cumin oil varied considerably, reaching 16.96% during the last storage stage periods at 4 °C, which is the highest value among the analyzed species. Therefore, black cumin seed oil also experienced the greatest induction-period decrease (74.11%) after 2 storage months. In this case, high peroxide levels indicated that the oils were unstable and they influenced the development of bad flavors.

Examining the various fat concentrations in the samples of seed oil cakes revealed statistically significant and observable species-specific differences. The milk thistle contained the least amount of fat at 8.24%, while black cumin contained the most at 23.24%.

Significant variations in fatty acid composition occurred during storage. In terms of polyunsaturated fatty acids, more substantial variations in fatty acid composition were identified. The content of linoleic, linolelaidic, linolenic, and arachidonic acids changed significantly.

As only storage period was assessed as the main factor for the chemical composition change of the assessed seed oils, additional criteria should be included, such as storage temperature, humidity or susceptibility to light.

## Figures and Tables

**Table 1:** Physicochemical properties of seeds oils.

Parameter	Milk Thistle	Safflower	Black Cumin
Acidity (% of oleic acid)			
Before storage	0.88 ± 0.04 ^a,A,^*	2.52 ± 0.01 ^b,A^	10.26 ± 0.77 ^c,A^
2 months after storage	0.87 ± 0.04 ^a,A^	2.60 ± 0.09 ^b,A^	15.91 ± 0.27 ^c,B^
4 months after storage	1.60 ± 0.03 ^a,B^	3.30 ± 0.07 ^b,B^	16.97 ± 0.58 ^c,C^
Acid value (mg KOH/g oil)			
Before storage	1.74 ± 0.07 ^a,A^	5.02 ± 0.03 ^b,A^	20.41 ± 1.53 ^c,A^
2 months after storage	1.73 ± 0.08 ^a,A^	5.17 ± 0.19 ^b,A^	31.66 ± 0.53 ^c,B^
4 months after storage	3.18 ± 0.06 ^a,B^	6.58 ± 0.15 ^b,B^	33.75 ± 0.58 ^c,C^
Peroxide value, meq/kg			
Before storage	0.56 ± 0.04 ^a,A^	3.49 ± 0.76 ^b,A^	143.1 ± 1.22 ^c,A^
2 months after storage	0.94 ± 0.06 ^a,B^	3.03 ± 0.06 ^b,A^	174.68 ± 32.83 ^c,B^
4 months after storage	1.48 ± 0.34 ^a,C^	5.49 ± 0.58 ^b,B^	59.43 ± 0.30 ^c,C^
Induction period, hours			
Before storage	2.21 ± 0.24 ^b,A^	1.45 ± 0.08 ^a,A^	6.49 ± 0.13 ^c,A^
2 months after storage	2.78 ± 0.30 ^b,B^	2.84 ± 0.91 ^b,B^	1.68 ± 0.07 ^a,C^
4 months after storage	1.99 ± 0.03 ^b,A^	0.76 ± 0.09 ^a,C^	4.01 ± 0.13 ^c,B^
Phenolic compounds, mg GAE 100 g^−1^			
Before storage	58.05 ± 0.07 ^a,B^	133.35 ± 0.03 ^b,A^	140.19 ± 0.11 ^c,A^
4 months after storage	37.40 ± 0.02 ^a,A^	39.06 ± 0.01± 0.00 ^c,B^	107.96 ± 0.01 ^b,B^

Lowercase letters “^a,b,c^” indicates significant differences when *p* < 0.05 (i.e., 95% reliability) between species. Hence, uppercase letters “^A,B,C^” depict significant differences of storage time when *p* < 0.05 (i.e., 95% reliability).

**Table 3:** Percentage of fatty acid groups in seed oils from a total fatty acid content, %.

Fatty Acids	Milk Thistle	Safflower	Black Cumin
SFA			
Before storage	33.69 ± 0.51 ^A,a,^*	32.22 ± 0.41 ^A,a^	24.68 ± 0.48 ^A,b^
4 months after storage	30.64 ± 0.20 ^B,b^	34.01 ± 0.13 ^A,a^	25.31 ± 0.22 ^A,c^
MUFA			
Before storage	28.08 ± 0.03 ^B,c^	30.85 ± 0.03 ^A,a^	33.52 ± 0.15 ^B,b^
4 months after storage	29.75 ± 0.33 ^A,c^	31.45 ± 0.14 ^Ab^	35.55 ± 0.02 ^A,a^
PUFA			
Before storage	38.23 ± 0.48 ^A,b^	36.93 ± 0.44 ^A,b^	41.79 ± 0.33 ^A,a^
4 months after storage	39.61 ± 0.13 ^A,a^	34.54 ± 0.01 ^B,b^	39.44 ± 0.19 ^B,a^
Omega-3 acids			
Before storage	1.00 ± 0.04 ^A,c^	1.38 ± 0.02 ^A,b^	2.63 ± 0.04 ^A,a^
4 months after storage	0.51 ± 0.02 ^B,c^	0.74 ± 0.02 ^B,b^	2.71 ± 0.03 ^A,a^
Omega-6 acids			
Before storage	37.23 ± 0.72 ^A,b^	35.54 ± 0.60 ^A,b^	39.88 ± 0.49 ^A,a^
4 months after storage	39.11 ± 0.20 ^A,a^	33.80 ± 0.00 ^A,c^	37.21 ± 0.26 ^A,b^
Omega-9 acids			
Before storage	19.89 ± 0.42 ^A,b^	14.73 ± 0.11 ^A,c^	25.11 ± 0.37 ^A,a^
4 months after storage	26.02 ± 0.50 ^B,a^	16.60 ± 0.20 ^B,b^	25.95 ± 0.22 ^A,a^
MUFA/PUFA			
Before storage	0.73	0.84	0.80
4 months after storage	0.75	0.91	0.90
PUFA/SFA			
Before storage	1.13	1.15	1.69
4 months after storage	1.29	1.02	1.56
n-6/n-3			
Before storage	37.23	25.75	15.16
4 months after storage	76.69	45.68	13.73
U/S			
Before storage	1.97	2.10	3.05
4 months after storage	2.26	1.94	2.95

* Lowercase letters “^a,b,c^” indicate significant differences when *p* < 0.05 (i.e., 95% reliability) between species. Hence, uppercase letters “^A,B,C^” depict significant differences in storage time when *p* < 0.05 (i.e., 95% reliability). SFA, saturated fatty acids; MUFA, monounsaturated fatty acids; PUFA, polyunsaturated fatty acids; U/S, unsaturated fatty acid/saturated fatty acid.

## Data Availability

Not applicable.

## Supplementary Materials

The following supporting information can be downloaded at: https://www.mdpi.com/article/10.3390/plants12061351/s1, Table S1: Possible compounds identified by an electronic nose in fixed seeds oils.
