# Supplementary material for: Quality Changes of Cold-Pressed Black Cumin (Nigella sativa L.), Safflower (Carthamus tinctorius L.), and Milk Thistle (Silybum marianum L.) Seed Oils during Storage
Source: Plants (Basel). 2023 Mar 17;12(6):1351. doi: 10.3390/plants12061351 (PMC10052079; doi:10.3390/plants12061351)
Supplement: Supplementary file 1 [file plants-12-01351-s001.zip › plants-2264995-supplementary.pdf]

Table S1. Possible matched compounds in oils.

KI MXT-5 a	KI MXT-1701 b	Possible Matched Compounds	Black cumin seed oil before storage	Black cumin seed oil after storage	Safflower seed oil before storage	Safflower seed oil after storage	Milk thistle before storage	Milk thistle after storage
434	493	Acetaldehyde		+		+		
437	564	Ethanol					+	
478	586	Propan-2-one					+	
484	559	Dimethyl sulfide	+				+	
522	626	2-methylpropanal	+	+				
571	683	Butane-2,3-dione				+	+	
614	695	Chloroform					+	
587	685	Butan-2-one	+	+				
619	773	Acetic acid	+					+
628	735	1-Propanol, 2-methyl	+		+			+
651	718	Isopropyl acetate	+	+			+	
652	729	3-methylbutanal		+				
646	751	But-(E)-2-enal					+	
643	810	1-Hydroxy-2-propanone					+	
657	714	Benzene					+	
651	768	n-butanol	+	+	+		+	+
691	795	Pentan-2-ol					+	
700	700	Heptane					+	
699	740	Trichloroethylene					+	
709	766	Ethyl propanoate	+	+	+			+
698	788	2,3-Pentanedione	+	+	+	+	+	+
770	968	2-methylpropanoic acid				+		
754	837	2-Methylpentanal					+	
777	840	Methyl 2-methylbutanoate	+	+	+			
755	889	Propanoic acid		+			+	+
755	889	Propionic acid			+			
756	915	Pyrrole			+		+	+
746	943	Propylenglycol					+	
777	840	Methyl 2-methylbutanoate					+	
769	888	(E)-2-penten-1-ol					+	
775	950	Formamide,N,N-dimethyl-					+	
800	800	Octane					+	
810	879	Butyl acetate	+		+	+		
816	970	Butanoic acid				+		

827	972	furfural				+		
849	907	Ethyl 2-methylbutyrate			+	+		+
852	960	(Z)-3-hexen-1-ol			+			
865	1027	3-methylbutanoic acid	+					
874	941	Isoamyl acetate	+	+				
883	905	Dibutyl ether					+	
888	969	3-Heptanone	+		+	+		
888	983	2-Heptanone		+				
898	953	Pentane, 3,3-diethyl-					+	
902	1084	Pentanoic acid						+
906	993	2,5-Dimethylpyrazine		+			+	
900	988	2,3-Dimethylpyrazine						+
920	995	3-Hexen-1-ol, formate, (Z)-					+	
897	986	Heptanal	+					
915	1005	2,3-dimethylpyrazine				+		
920	991	2,6-Dimethylpyrazine	+		+			
924	994	Ethylpyrazine					+	
932	945	1 R-(+)-alpha-pinene	+	+				
936	950	1S-()-a-pinene	+		+	+	+	
984	1007	()-beta-Pinene	+	+	+		+	
987	1086	2-Octanone					+	
990	1186	Hexanoic acid			+		+	
1017	1089	Alpha-Terpinene					+	
1023	1135	Acetylpyrazine			+			
998	1090	Octanal	+					
1025	1073	p-Cymene		+				
1033	1061	Limonene	+	+				
1032	1085	1,8-cineole					+	+
1024	1141	2-acetylthiazole					+	
1023	1135	acetylpyrazine						+
1060	1089	Gamma-Terpinene	+	+				
1065	1176	1-Octanol	+	+				
1093	1183	Nonan-2-one	+	+				
1100	1194	n-nonanal		+				
1066	1208	Acetophenone	+					
1148	1248	Citronellal	+	+				
1149	1306	Benzoic acid			+			+
1165	1277	1-nonanol			+			
1164	1288	Benzyl acetate	+	+				

1182	1321	p-methylacetophenone					+	
1200	1317	Methyl salicylate				+		+
1216	1339	(E,E)-2,4-Nonadienal					+	
1291	1483	Thymol					+	
1206	1293	Decanal			+			
1200	1317	Methyl salicylate			+			
1228	1344	Citronellol	+					
1261	1475	4-Octanolide	+					
1283	1390	Anethole	+	+				
1282	1430	4-ethylguaiacol			+			
1365	1483	trans-2-Undecenal				+	+	
1363	1563	gamma-nonalactone					+	
1427	1490	Methyl undecanoate					+	
1420	1662	Vanillin					+	
1426	1552	Alpha-ionone					+	

[a] Kovats index for non-polar column; [b] Kovats index for slightly polar column.
